# Unveiling Heart Health: A National Sociodemographic Exploration of Cardiovascular Risk Factors and Prevention Strategies in Saudi Arabia

**DOI:** 10.7759/cureus.92908

**Published:** 2025-09-22

**Authors:** Razan Adel Gobis, Lin Mazen Jolha, Hala Algazzawi, Abdulelah K Alqawlaq, Leen Naife Subahi, Lana Naife Subahi, Muhammad S Reihan

**Affiliations:** 1 Department of General Medicine, Batterjee Medical College, Jeddah, SAU; 2 Department of Medicine and Surgery, Batterjee Medical College, Jeddah, SAU; 3 Department of Internal Medicine, Batterjee Medical College, Jeddah, SAU; 4 Department of Cardiology, Faculty of Medicine, Al-Azhar University, Damietta, SAU

**Keywords:** awareness, cardiovascular disease, cardiovascular risk factors, knowledge, practice, primary prevention

## Abstract

Background

Cardiovascular diseases (CVDs) stand out as the chief cause of mortality and morbidity worldwide and in Saudi Arabia due to a variety of causes and risk factors, some of which are modifiable, and others are non-modifiable. Although many studies have been conducted in Saudi Arabia to estimate the incidence of CVD cases, there has not been a published study that assessed the public knowledge, attitude, and practice (KAP) of CVD, its risk factors, and the primary interventions that can be carried out to alleviate this crucial health burden.

Methodology

A cross-sectional observational study was conducted among the different regions of Saudi Arabia, using a questionnaire adapted from a previous study, aiming to assess the public’s KAP to identify the misconceptions and cover up the gaps among the general Saudi population, contributing to the development of proper awareness raising and educational programs to educate the public about it.

Results

Out of 1,836 participants, good KAP values were 60.0%, 26.6%, and 9.5%, respectively. Calculated p-values vary among different sociodemographic groups.

Conclusion

The study reveals moderate knowledge among participants, accompanied by a low attitude and practice, highlighting the need for the development of targeted public health strategies to enhance their knowledge, strengthen their attitude, and encourage them to practice behaviors that support their cardiovascular health.

## Introduction

Cardiovascular diseases (CVDs) are the leading cause of death globally, with a yearly estimated death toll of 17.9 million [1]. The term CVDs is a broad term used to group diseases affecting the heart and vascular system, which can be further subdivided into four main categories: coronary artery disease, cerebrovascular disease, peripheral arterial disease, and aortic atherosclerosis [2].

The major cause of death in Saudi Arabia is CVD, with it being responsible for more than 45.7% of all mortality cases [3-5]. The yearly national mortality is 294 deaths per 100,000 people, with an extra 3,702 people dying from non-fatal CVD [4,5]. A review article published in the Journal of the Saudi Heart Association states that in 2016, a total of 201,300 cases of CVD were recorded, out of which 149,600 cases were diagnosed with ischemic heart disease, and 51,700 cases were diagnosed with cerebrovascular diseases [6]. According to a 2017 national perspective research, the prevalence of CVD in Saudis aged 15 and above is 1.6%, with males (1.9%) having a greater rate of incidence than females (1.4%) [7]. The prevalence of CVD in Saudi Arabia is expected to grow to affect about 480,000 people by 2035 [8].

The increasing prevalence of CVD in Saudi Arabia can be attributed to an increase in modifiable risk factors due to urbanization, shift in habits, and socioeconomic positions [7,9]. These changes extended to affect the lifestyle of the Saudi population as it contributed to a lack of adequate nourishment and exercise with a rise in sedentary behavior [9-12]. The Prospective Urban Rural Epidemiology (PURE-Saudi) study also revealed a significant prevalence of unhealthy lifestyle habits and CVD risk factors in the adult Saudi population, with rates greater in rural than urban areas [13].

Many studies have been conducted to quantify the prevalence and incidence of CVD in Saudi Arabia, but there is a lack of research assessing the Saudi public’s knowledge, attitude, and practice (KAP) regarding CVD and its risk factors and primary prevention. Identifying the gaps and misconceptions in knowledge will allow for better development of targeted educational programs and interventions to help decrease the rise of CVDs.

This study aims to assess the knowledge, attitudes, and practices of the Saudi population regarding CVD risk factors and their primary prevention.

## Materials and methods

Study design

This study employed a qualitative online cross-sectional survey conducted throughout March 2025, distributed to all five regions of the Kingdom of Saudi Arabia. The main aim of the study was to measure the knowledge, attitude, and practice of the general Saudi population toward the risk factors of CVD and primary prevention using a previously validated bilingual survey.

Population

The study targeted Saudi citizens and residents over the age of 18 who speak and understand the language of the survey, English or Arabic. Those who were under 18 years old, did not reside in Saudi Arabia, adults with poor cognitive functions, or who refused to participate were excluded from the study.

Sample size

The minimum sample size was calculated from the Raosoft website (Raosoft, Inc., Seattle, WA), considering a level of confidence of 95%, an expected prevalence of 50% and a precision of 0.05, which was found to be 384.

To obtain a sample size of 32,175,224 with a confidence level of 95% and a margin of error of 5%, the sample size calculation was performed using Slovin’s formula n = (Zα/2)^2^ * P(1 - P) / d^2^. In this equation, n represents the calculated sample size. For a 95% confidence level, the critical value Zα/2 is approximately 1.96. Assuming an estimated prevalence of P as 0.5 (50%), and a margin of error, d, set at 0.05 (5%), the formula was applied. Substituting these values, the calculation yielded n = (1.96)^2^ * 0.5(1 - 0.5) / 0.05^2^, resulting in a sample size estimate of approximately 384. Therefore, to achieve a sample size of 32,175,224 with a confidence level of 95% and a margin of error of 5%, the calculated sample size would still be approximately 384, as it is not directly proportional to the population size.

Survey development

The questionnaire is from a previously published and validated survey created in the UAE (Appendices) [14], with modifications of the sociodemographics to suit the sample population. It was designed and developed by a multidisciplinary team, and the evaluation tool underwent a pilot study to assess the ease of understanding, potential pitfalls in the methodology, study design, data collection techniques, and the time required to complete it. The survey instrument consists of 46 items. The first section of the questionnaire collects sociodemographic data: age, nationality, marital status, employment status, academic level, monthly income, and health-related history. The remaining sections of the questionnaire focus on KAP toward CVD. There are 16 questions assessing the population’s knowledge of CVD risk factors, nine assessing the attitude, and 11 assessing their practice. Each of the questions is equally scored (one point for each correct answer and zero otherwise), and these scores are then summed across all the questions. Participants are categorized according to their score.

Data collection

The general public was invited to participate in the study through online platforms, such as WhatsApp (Meta Platforms, Menlo Park, CA) and Telegram (Pavel Durov and Nikolai Durov, Dubai, UAE), using convenience sampling. The online questionnaire provided information about the survey with eligibility questions at the beginning to ensure only adult residents in Saudi Arabia could participate. The questionnaire was available in both English and Arabic and was distributed online through Google Forms (Google, Inc., Mountain View, CA).

Data analysis

The data underwent careful elimination of the excluded participants, followed by coding and analysis in IBM Statistical Package for Social Sciences (SPSS) version 26 (IBM Corp., Armonk, NY). Descriptive (frequencies, percentages, mean, and standard deviation) and inferential statistical analysis (chi-square test) were used to explore the relation and associations among the variables in this study. This study will determine the level of awareness and understanding among the Saudi general population about CVD risk factors and their primary prevention.

The knowledge section contains 16 questions with true, false, and "I don't know." A score of 0-16 is given according to the selection of the correct statements, with consideration of a poor knowledge score of 0-5, a fair knowledge score of 6-10, and a good knowledge score of 11-16.

The attitude section contains nine questions with strongly disagree (-2) to strongly agree (+2). A score of 0-18 is calculated with consideration of a poor attitude score of 0-6, a fair attitude score of 7-12, and a good attitude score of 13-18.

The practice section contains 11 questions with never (0), seldom (1), frequently (2), and always (3). A score of 0-33 is calculated according to the participants' choice selection, with consideration of a poor practice of 0-10, fair practice of 11-22, and good practice of 23-33. Negative statement questions were taken into consideration during the coding process.

Ethical considerations

This study received ethical approval from the Ethics and Scientific Committees of Batterjee Medical College under Research Code RES-2025-0011, granted on February 27, 2025. All participants provided informed consent, confirming their voluntary involvement. No personally identifiable information was collected, and only the research team had access to the data.

## Results

The final number of voluntary participants in the study was 1,836 individuals. The greater proportion of these participants were between the ages of 18 and 25 years, with a mean age of 29.18 ± 10.15 years. The majority of whom were females, 1,166 (63.5%), Saudi nationals, 1,428 (77.8%), and single, 1,022 (55.7%). The preponderance of the participants resided in the Northern region, 497 (27.1%), Central, 441 (24.0%), and Western, 390 (21.2%). 

As for the employment status, the greater portion were not employed, 1,078 (58.7%), students, 624 (34.0%), and housewives, 434 (23.6%). A limited group of participants worked in education 299 (16.3%), and a minority worked in corporate jobs 158 (8.6%) or engineering 135 (7.4%). Almost all participants had earned a university/postgraduate degree, 1,412 (76.9%), and a moderate monthly income, 1,028 (56.0%). Defined as “just enough,” followed by 598 (32.6%) of participants who did not earn enough.

Among the participants, 1,028 (56.0%) were non-smokers, 598 (32.6%) were smokers, and 210 (11.4%) were ex-smokers. A total of 1,214 participants (60.2%) reported no medical history, while 265 (13.1%) had diabetes mellitus, 195 (9.7%) had hypertension, and 183 (9.1%) had high cholesterol. In addition, 503 participants (27.4%) reported a family history of CVD (Table 1).

**Table 1:** Descriptive analysis of demographics ^α^Other careers: lawyers, freelancers, tailors, artists, hairdressers, and pilots. ^β^Multiple responses are allowed. ^γ^Other medical history: anemia, asthma, hypo/hyperthyroidism, irritable bowel syndrome, inflammatory bowel disease, psoriasis, insulin resistance, eczema, valvular heart disease, chronic kidney disease, rheumatic fever, vitamin D deficiency, depression and panic attacks, systemic lupus erythematosus, and Sjögren’s syndrome.

Variable	Frequency N = 1,836 (%)
Gender	
Male	670 (36.5)
Female	1,166 (63.5)
Age	
18-25	857 (46.7)
26-35	518 (28.2)
36-45	291 (15.8)
≥46	170 (9.3)
Nationality	
Saudi	1,428 (77.8)
Non-Saudi	408 (22.2)
Region	
Northern	497 (27.1)
Southern	307 (16.7)
Eastern	201 (10.9)
Western	390 (21.2)
Central	441 (24)
Marital status	
Single	1,022 (55.7)
Married	783 (42.6)
Divorced/widowed	31 (1.7)
Employment status	
Employed/self-employed	735 (40)
Retired	23 (1.3)
Not employed	1,078 (58.7)
Career	
Student	624 (34)
Engineer	135 (7.4)
Education	299 (16.3)
Military	43 (2.3)
Corporate job	158 (8.6)
Trading	109 (5.9)
Housewife	434 (23.6)
Other^α^	34 (1.9)
Monthly income	
Low (not enough)	598 (32.6)
Moderate (just enough)	1,028 (56)
High (enough and saving)	210 (11.4)
Education	
Middle/high school	424 (23.1)
University/postgraduate	1,412 (76.9)
Smoker	
Yes	598 (32.6)
No	1,028 (56)
Ex-smoker	210 (11.4)
Medical history^β^	
Hypertension	195 (9.7)
Diabetes mellitus	265 (13.1)
High cholesterol	183 (9.1)
Ischemic heart disease	59 (2.9)
Other^γ^	100 (5)
None	1,214 (60.2)
Family history of CVD	
Yes	503 (27.4)
No	1,333 (72.6)

Knowledge of cardiovascular risk factors

**Figure 1:**
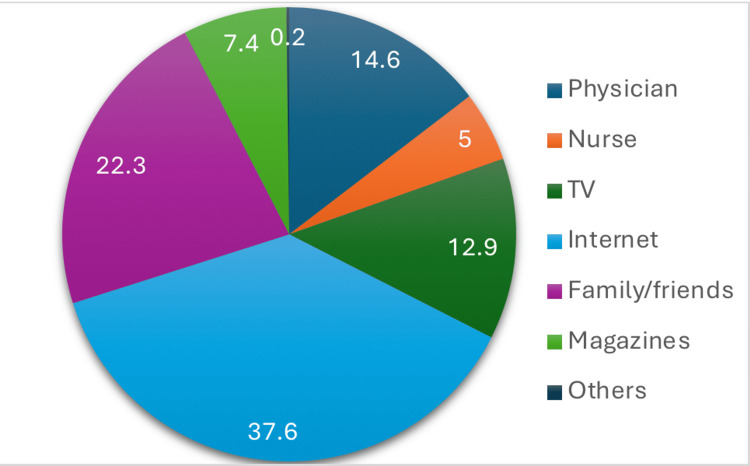
Participants’ knowledge source about cardiovascular disease (CVD).

The participants’ knowledge of cardiovascular risk factors showed considerable variation across different sociodemographic groups. Among genders, the differences were of little variation, with females 711 (61.0%) vs. males 390 (58.2%), although this was not a statistically significant difference with a calculated p-value of (p = 0.314). Differences between age groups exhibited notable variation in knowledge, with ages between 18 and 25 years showing “good knowledge” 504 (58.8%), and the eldest age group, ≥46 years, showing 95 (55.9%), although again, these differences were not statistically significant with a p-value of (p = 0.266). On the other hand, the difference between Saudi and non-Saudi respondents was found to be considerably variable, with non-Saudis 279 (68.4%) vs. Saudis 822 (57.6%) in terms of knowledge. This notable variation may be attributed to the differences in education or access to healthcare information. Employment status also played a role, with employed/self-employed individuals having 442 (60.1%) “good knowledge,” while those with higher education, university/postgraduate, demonstrated the highest knowledge levels at 851 (60.3%). Table 2 shows the sociodemographic factors associated with knowledge of cardiovascular risk factors. Figure 1 demonstrates the different sources and references that participants used to obtain knowledge about CVD risk factors. The greater part of 690 (37.6%) of participants use the internet as a primary source for their information, followed by 409 (22.3%) of whom their source was family and friends, and 268 (14.6%) from physicians. Some other notable sources were from television, 237 (12.9%), and magazines, 136 (7.4%). These results highlight the growing influence of digital platforms in health education, while also emphasizing the importance of informal networks and healthcare professionals.

**Table 2:** Sociodemographic factors associated with knowledge and attitude to cardiovascular disease (CVD) *χ² = chi-square test. *p ≤ 0.05 is considered statistically significant. ^α^Multiple responses are allowed. ^β^Other medical history: anemia, asthma, hypo/hyperthyroidism, irritable bowel syndrome, inflammatory bowel disease, psoriasis, insulin resistance, eczema, valvular heart disease, chronic kidney disease, rheumatic fever, vitamin D deficiency, depression and panic attacks, systemic lupus erythematosus, and Sjögren’s syndrome.

Variable	Knowledge					Attitude				
	Good N (%)	Fair N (%)	Poor N (%)	Test value (χ²)	p-value	Good N (%)	Fair N (%)	Poor N (%)	Test value (χ²)	p-value
Gender										
Male	390 (58.2)	226 (33.7)	54 (8.1)	2.316	0.314	175 (26.1)	253 (37.8)	242 (36.1)	1.460	(0.482)
Female	711 (61)	380 (32.6)	75 (6.4)			314 (26.9)	463 (39.7)	389 (33.4)		
Age										
18-25	504 (58.8)	288 (33.6)	65 (7.6)	7.634	0.266	234 (27.3)	339 (39.6)	284 (33.1)	28.422	(0.000*)
26-35	313 (60.4)	175 (33.8)	30 (5.8)			132 (25.5)	214 (41.3)	172 (33.2)		
36-45	189 (64.9)	85 (29.2)	17 (5.8)			78 (26.8)	103 (35.4)	110 (37.8)		
≥46	95 (55.9)	58 (34.1)	17 (10)			45 (26.5)	60 (35.3)	65 (38.2)		
Nationality										
Saudi	822 (57.6)	491 (34.4)	115 (8.1)	19.535	0.000*	394 (27.6)	539 (37.7)	495 (34.7)	4.959	(0.084)
Non-Saudi	279 (68.4)	115 (28.2)	14 (3.4)			95 (23.3)	177 (43.4)	136 (33.3)		
Region										
Northern	301 (60.6)	159 (32)	37 (7.4)	15.158	0.056	120 (24.1)	184 (37.0)	193 (38.8)	37.426	(0.000*)
Southern	171 (55.7)	109 (35.5)	27 (8.8)			71 (23.1)	102 (33.2)	134 (43.6)		
Eastern	128 (63.7)	53 (26.4)	20 (10)			49 (24.4)	79 (39.3)	73 (36.3)		
Western	232 (59.5)	142 (36.4)	16 (4.1)			123 (31.5)	150 (38.5)	117 (30)		
Central	269 (61)	143 (32.4)	29 (6.6)			126 (28.6)	201 (45.6)	114 (25.9)		
Marital status										
Single	600 (58.7)	352 (34.4)	70 (6.8)	2.192	0.701	293 (28.7)	414 (40.5)	315 (30.8)	28.422	(0.000*)
Married	482 (61.6)	244 (31.2)	57 (7.3)			196 (25)	292 (37.3)	295 (37.7)		
Divorced/widowed	19 (61.3)	10 (32.3)	2 (6.5)			0	10 (32.3)	21 (67.7)		
Employment status										
Employed/self-employed	442 (60.1)	234 (31.8)	59 (8)	2.495	0.645	187 (25.4)	262 (35.6)	286 (38.9)	15.774	(0.003*)
Retired	13 (56.5)	8 (34.8)	2 (8.7)			7 (30.4)	5 (21.7)	11 (47.8)		
Not employed	646 (59.9)	364 (33.8)	68 (6.3)			295 (27.4)	449 (41.7)	334 (31)		
Career										
Student	376 (60.3)	214 (34.3)	34 (5.4)	31.883	0.004*	166 (26.6)	264 (42.3)	194 (31.1)	23.093	(0.059)
Engineer	66 (48.9)	55 (40.7)	14 (10.4)			38 (28.1)	55 (40.7)	42 (31.1)		
Education	177 (59.2)	106 (35.5)	16 (5.4)			64 (21.4)	112 (37.5)	123 (41.1)		
Military	26 (60.5)	14 (32.6)	3 (7)			9 (20.9)	12 (27.9)	22 (51.2)		
Corporate job	91 (57.6)	48 (30.4)	19 (12)			41 (25.9)	56 (35.4)	61 (38.6)		
Trading	83 (76.1)	18 (16.5)	8 (7.3)			29 (26.6)	41 (37.6)	39 (35.8)		
Housewife	261 (60.1)	140 (32.3)	33 (7.6)			129 (29.7)	164 (37.8)	141 (32.5)		
Other	21(61.8)	11 (32.4)	2 (5.9)			13 (38.2)	12 (35.3)	9 (26.5)		
Monthly income										
Low (not enough)	344 (57.5)	220 (36.8)	34 (5.7)	17.467	0.002*	150 (25.1)	260 (43.5)	188 (31.4)	11.519	(0.021*)
Moderate (just enough)	634 (31.7)	326 (31.7)	68 (6.6)			291 (28.3)	380 (37)	357 (34.7)		
High (enough and saving)	123 (58.6)	60 (28.6)	27 (12.9)			48 (22.9)	76 (36.2)	86 (41)		
Education										
Illiterate/primary	0	0	0		0.836	0	0	0		(0.055)
Middle/high school	250 (59)	145 (34.2)	29 (6.8)	0.357		138 (32.5)	186 (43.9)	100 (23.6)	5.809	
University/postgraduate	851 (60.3)	461 (32.6)	100 (7.1)			493 (34.9)	530 (37.5)	386 (27.5)		
Smoker										
Yes	123 (50.2)	81 (33.1)	41 (16.7)	44.879	0.000*	40 (16.3)	75 (30.6)	130 (53.1)	48.224	(0.000*)
No	922 (61.1)	504 (33.4)	82 (5.4)			426 (28.2)	614 (40.7)	468 (31)		
Ex-smoker	56 (67.5)	21 (25.3)	6 (7.2)			23 (27.7)	27 (32.5)	33 (39.8)		
Medical history^α^										
Hypertension	112 (57.4)	70 (35.9)	13 (6.7)	8.273	0.772	60 (30.8)	49 (25.1)	86 (44.1)	43.731	(0.000*)
Diabetes mellitus	153 (57.7)	97 (36.6)	15 (5.7)			68 (25.7)	101 (38.1)	96 (36.2)		
High cholesterol	107 (58.5)	63 (34.4)	13 (7.1)			31 (16.9)	66 (36.1)	86 (47)		
Ischemic heart disease	38 (64.4)	14 (23.7)	7 (11.9)			14 (23.7)	27 (45.8)	18 (30.5)		
Other^β^	56 (56)	35 (35)	9 (9)			26 (26)	41 (41)	33 (33)	33	
None	728 (60)	402 (33.1)	84 (6.9)			333 (27.4)	487 (40.1)	394 (32.5)	394	
Family history of CVD										
Yes	318 (63.2)	145 (28.8)	40 (8)	5.735	0.057	125 (24.9)	223 (44.3)	155 (30.8)	8.430	(0.015*)
No	783 (58.7)	461 (34.6)	89 (6.7)			364 (27.3)	493 (37)	476 (35.7)		

Attitude toward cardiovascular risk factors

Attitude toward cardiovascular risk factors demonstrated a considerable variation in sociodemographic differences, especially the discrepancy of results among males and females. Results showed more equitable distribution of attitudes among males, with 253 (37.8%) showing a “fair attitude” and 242 (36.1%) showing a “poor attitude.” On the other hand, females showed 463 (39.7%) “fair attitudes” and 389 (33.4%) “poor attitudes.” Age discrepancies were not of meaningful significance statistically on attitudes (p = 0.535). The 26-35 years age group, however, had the highest percentage, 214 (41.3%), of “fair attitudes” and the second lowest percentage, 172 (33.2%), of the “poor attitudes category” after the 18-25 years age group with a “poor attitudes category” of 284 (33.1%). Attitudes were also strongly influenced by marital status (p = 0.000). Results demonstrated that the highest percentage of “good attitude” and “fair attitude,” 293 (28.7%) and 414 (40.5%), respectively, were among single individuals compared to married individuals, with the highest “poor attitude” category of 295 (37.7%). This may be attributed to greater health awareness in single individuals. Findings among smokers and non-smokers demonstrated significant divergence, with smokers 130 (53.1%) having a poor attitude vs. non-smokers 468 (31.0%). These findings highlight the need to target smokers in public health campaigns and advertisements focused on improving attitudes toward cardiovascular health.

Practice toward cardiovascular risk factors

Results demonstrated females had the highest share, 764 (65.5%), in the “fair practice” category compared to 416 (62.1%) in males (p = 0.023). Participants in the youngest age group, 18-25 years, 76 (8.9%), had less “good practice” than the elderly group, ≥46 years, 26 (15.3%) (p = 0.011). Occupation showed that students, 57 (9.1%), had the lowest “good practice” and highest, 189 (30.3%), “poor practice” (p = 0.000). Individuals with higher monthly income were associated with better practice, 30 (14.3%, p = 0.022). Smokers, 79 (32.2%), again showed “poor practice” in comparison with non-smokers, 388 (25.7%, p = 0.002). In terms of people with medical histories, hypertensive individuals had better overall practice, 25 (12.8%, p = 0.006). Table 3 presents sociodemographic factors associated with practices toward cardiovascular risk factors.

**Table 3:** Sociodemographic factors associated with practice of cardiovascular disease (CVD) *χ² = chi-square test. *p ≤ 0.05 is considered statistically significant. ^α^Multiple responses are allowed. ^β^Other medical history: anemia, asthma, hypo/hyperthyroidism, irritable bowel syndrome, inflammatory bowel disease, psoriasis, insulin resistance, eczema, valvular heart disease, chronic kidney disease, rheumatic fever, vitamin D deficiency, depression and panic attacks, systemic lupus erythematosus, and Sjögren’s syndrome.

Variable	Practice			Test value (χ²)	p-value
	Good N (%)	Fair N (%)	Poor N (%)		
Gender					
Male	80 (11.9)	416 (62.1)	174 (26)	7.567	(0.023*)
Female	94 (8.1)	764 (65.5)	308 (26.4)		
Age					
18-25	76 (8.9)	536 (62.5)	245 (28.6)	16.594	(0.011*)
26-35	52 (10)	329 (63.5)	137 (26.4)		
36-45	20 (6.9)	206 (70.8)	65 (22.3)		
≥46	26 (15.3)	109 (64.1)	35 (20.6)		
Nationality					
Saudi	145 (10.2)	901 (63.1)	382 (26.8)	5.094	(0.078)
Non-Saudi	29 (7.1)	279 (68.4)	100 (24.5)		
Region					
Northern	44 (8.9)	329 (66.2)	124 (24.9)	6.818	(0.556)
Southern	28 (9.1)	188 (61.2)	91 (29.6)		
Eastern	26 (12.9)	120 (59.7)	55 (27.4)		
Western	33 (8.5)	255 (65.4)	102 (26.2)		
Central	43 (9.8)	288 (65.3)	110 (24.9)		
Marital status					
Single	103 (10.1)	646 (63.2)	273 (26.7)	5.242	(0.263)
Married	71 (9.1)	514 (65.6)	198 (25.3)		
Divorced/widowed	0	20 (64.5)	11 (35.5)		
Employment status					
Employed/self-employed	71 (9.7)	479 (65.2)	185 (25.2)	3.271	(0.514)
Retired	2 (8.7)	18 (78.3)	3 (13)		
Not employed	101 (9.4)	683 (63.4)	294 (27.3)		
Career					
Student	57 (9.1)	378 (60.6)	189 (30.3)	69.979	(0.000*)
Engineer	15 (11.1)	86 (63.7)	34 (25.2)		
Education	25 (8.4)	221 (73.9)	53 (17.7)		
Military	12 (27.9)	31 (72.1)	0		
Corporate job	14 (8.9)	100 (63.3)	44 (27.8)		
Trading	7 (6.4)	54 (49.5)	48 (44)		
Housewife	44 (10.1)	288 (66.4)	102 (23.5)		
Other	0 (0)	22 (64.7)	12 (35.3)		
Monthly income					
Low (not enough)	41 (6.9)	389 (65.1)	168 (28.1)	11.493	(0.022*)
Moderate (just enough)	103 (10)	660 (64.2)	265 (25.8)		
High (enough and saving)	30 (14.3)	131 (62.4)	49 (23.3)		
Education					
Illiterate/primary	0	0	0	0.141	(0.932)
Middle/high school	39 (9.2)	271 (63.9)	114 (26.9)		
University/postgraduate	135 (9.6)	909 (64.4)	368 (26.1)		
Smoker					
Yes	29 (11.8)	137 (55.9)	79 (32.2)	17.162	(0.002*)
No	143 (9.5)	977 (64.8)	388 (25.7)		
Ex-smoker	2 (2.4)	66 (79.5)	15 (18.1)		
Medical history^α^					
Hypertension	25 (12.8)	131 (67.2)	39 (20)	27.198	(0.006*)
Diabetes mellitus	13 (4.9)	193 (72.8)	59 (22.3)		
High cholesterol	20 (10.9)	116 (63.4)	47 (25.7)		
Ischemic heart disease	7 (11.9)	41 (69.5)	11 (18.6)		
Other^β^	5 (5)	60 (60)	35 (35)		
None	116 (9.6)	769 (63.3)	329 (27.1)		
Family history of CVD					
Yes	56 (11.1)	325 (64.6)	122 (24.3)	3.071	(0.215)
No	118 (8.9)	855 (64.1)	360 (27)		

## Discussion

Summary of key findings

A national cross-sectional survey was conducted to measure the KAP of CVD risk factors and primary prevention among 1,836 Saudi participants. A good level of knowledge, attitude, and health care practices was demonstrated by 1,101 (60.0%), 488 (26.6%), and 174 (9.5%) of the respondents, respectively. A large differential between knowledge and behavior underscores a major public health challenge in translating awareness into disease prevention.

**Figure 2:**
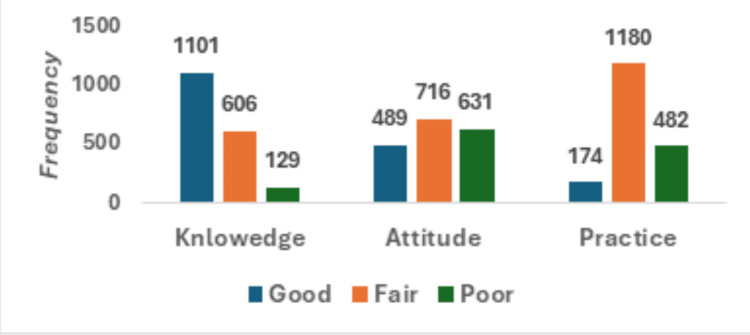
Knowledge, attitude, and practice toward cardiovascular disease (CVD) among the total study population (N = 1,846). The data represent overall frequencies.

Figure 2 shows the distribution of knowledge, attitude, and practice level, and it shows that 1,101 (60%) students had good knowledge, 174 (9.5%) good practice, and 1,180 (64.3%) fair practice. According to these findings, we underscore a profound disconnect between awareness and actual behavioral change in the Saudi population. These findings highlight a critical gap between awareness and behavioral change in the Saudi population, and this study highlights the need for strategies that convert knowledge into sustained preventive actions.

Comparison with previous studies

In our findings, there were moderate levels of knowledge 64.5% and relatively good attitude and practice scores compared with national Saudi samples, which is consistent with reports by Alaamri and Naser [15]. In contrast, AlShehri et al. demonstrated a positive attitude in 87.3% of the subjects, which contrasts with the 26% in our group, indicating a marked regional variation [16]. However, the split between what we know and what we do turned out to be even deeper, according to the current studies.

A Riyadh study from Alduraywish et al. (2022) found that diabetic patients had better knowledge scores, particularly patients with higher education and income [17]. Basubrain et al. and Awad and Al-Nafisi have highlighted the extensive knowledge gaps of the public on symptoms and risk factors, particularly among smokers and low-income individuals [18,19]. Similar modest knowledge but poor practice of lifestyle changes was observed among the general populations in Kuwait and Lebanon when measured by KAP surveys [19,20]. A meta-analysis of Trejo et al. (2018) found that poor attitudes and practices of health knowledge in Saudi adults aged 15 to 25 years were similar to young adults in other global regions [21,22].

Awareness gaps and symptom recognition

However, even well-known risk factors, such as smoking and obesity, were poorly understood, as were rare factors (e.g., thyroid diseases, anemia), as in previous reports from Najran [16], Malaysia [23], and Kuwait [19]. This underscores the difficulty in operationalizing public education to guide comprehensive symptom recognition.

Sociodemographic predictors of KAP

KAP scores were statistically higher among educated, younger, single, and non-Saudi participants; such an association was in line with what was found for Riyadh, Lebanon, and Jordan [17,20]. Participants with lower scores were seniors, smokers, and people with low income, in accordance with local and international studies [15,24]. Furthermore, participants with higher income and those with professional occupations, such as trading, had higher knowledge scores, indicating that socioeconomic status and professional exposure affect cardiovascular health knowledge. Gender biases were also observed among females who had a good knowledge, but poor practice, as reported by Singapore and Jordan [25], although females demonstrated higher knowledge scores, males were more likely to report good preventive practices, possibly due to cultural and lifestyle constraints on women. Interestingly, males, 80 (11.9%), reported slightly better preventive practices compared to females, 94 (8.1%), although females exhibited better knowledge scores. Participants aged ≥ 46 years had lower knowledge, which may be linked to cognitive decline and reduced exposure to awareness campaigns. Conversely, single participants demonstrated better attitudes, likely due to a greater focus on personal health and self-care. In contrast, married participants tended to have lower attitude scores, which may reflect lifestyle patterns or family-related stressors that limit the adoption of preventive behavior. This finding may be indicative of a gender variation in knowledge but not practice, where even though women access and display awareness about health education, they struggle to convert that into behavior change due to maternal responsibilities, social roles, or exclusion from the decision-making process for health, as reported by previous regional reports from Singapore and Jordan.

Interpretation and behavioral gap

This agrees with other international trends, and it is consistent even though CVD knowledge available and the overall amount of preventive practice remain low, especially among young respondents, with lower educational attainment levels, and with Saudi participants. This fact ultimately leads to the non-cultivation of a healthy lifestyle, as young adults often do not see themselves at risk for this type of heart disease. Second, participants with less education may have low health literacy and incorrect beliefs about CVD risk factors. In addition to cultural eating and sedentary behaviors among Saudi participants, these behaviors widen the behavior gap. Motivation, lack of early symptoms, and financial limitations still represent significant obstacles, even in the presence of good knowledge, as reported in studies conducted in Kuwait, Lebanon, and the USA [19,24,26]. The post-awareness impenetrable barrier is still smoking, which validates the necessity of smoking cessation efforts in at-risk populations. In the context of public health promotion, the intensive smoking cessation programs should be given high priority because smokers are not only attitudinally more unhealthy but also less inclined to adopt preventive behaviors, even when equipped with knowledge. For example, the youth often lack motivation due to a low perceived risk, while low-income individuals face cost-related limitations, and women may encounter structural or social constraints that prevent active preventive behavior, which are consistent with previous data from Kuwait, Lebanon, and the United States [19,24,26]. Although the females demonstrated better knowledge levels, males were more likely to report good preventive practices, a finding consistent with regional studies from Singapore and Jordan, suggesting that social roles and decision-making limitations may hinder women’s ability to implement health knowledge.

Public health implications

However, outreach needs to be more than educational and consist of culturally sensitive behavior change interventions. Digital health tools and place-based programs may be used to fill such gaps more effectively, particularly in Southern Saudi Arabia. Preventive counseling in primary care should be strengthened, and a model of care that has been shown to be effective should be adopted in at least some centers in Saudi Arabia in cardiac rehabilitation studies [27]. Taking such grassroots approaches can help to improve motivation and ease barriers, particularly in the more marginalized or high-risk communities. In addition, community interventions, peer support, and e-health should be part of the public health strategy. Community-based screening programs should be expanded and paired with peer support groups to promote early detection and encourage lifestyle modification. There is a need for regionalized (including a focus on the South because of relatively low knowledge and preventive behavior) social-marketing campaigns.

Study limitations and strengthen

This study has several strengths. It comprised a large and geographically representative group (1,836 people from all regions of the Kingdom of Saudi Arabia) that made the sample more comprehensive on a nationwide level for the understanding of knowledge, attitudes, and practices toward CVD risk factors. Content validity and comparability are assured by the employment of an already validated and culturally adapted questionnaire. The data were collected in 2025, making the study contemporary, and the results are combined with a broad cross-section of regional and international literature, which increases the external validity. In addition, better knowledge of which sociodemographic factors predict is relevant to the correlates of awareness and prevention of CVD.

Despite its strengths, our study has some limitations. First, the use of self-reported questionnaires may have introduced recall bias and social desirability bias, particularly in reporting smoking, diet, or exercise behaviors. Second, our reliance on an online convenience sample may reduce generalizability, especially for older adults, individuals with lower education, or rural populations with limited internet access. Third, as a cross-sectional design, the study cannot establish causality between knowledge, attitudes, and practices. Finally, some sociodemographic categories (e.g., retirees, military, certain occupations) were underrepresented, which may limit subgroup analysis. These limitations should be considered when interpreting our results.

## Conclusions

Our study confirms a persistent gap between awareness and action in CVD prevention, particularly among youth, smokers, and low-income individuals. This underscores the need for targeted public health efforts, especially smoking cessation and youth-focused programs to translate knowledge into lasting heart-healthy behaviors. A key to achieving sustainable lifestyle changes for cardiovascular health is to address motivational and systemic barriers through community-based interventions.

## Appendices

Batterjee Medical Colleges (BMC) - Cardiovascular Disease (CVD) Survey

Demographic Data

Age: ___________

Gender: ☐ Male ☐ Female

Marital status: ☐ Single ☐ Married ☐ Widowed ☐ Divorced

Nationality: ☐ Saudi ☐ Non-Saudi

Region: ☐ Northern ☐ Eastern ☐ Southern ☐ Western ☐ Central

Employment: ☐ Employed/self-employed ☐ Retired ☐ Not employed

Career: ☐ Student ☐ Engineer ☐ Education ☐ Military ☐ Corporate job ☐ Trading ☐ Housewife ☐ Other: ________

Education: ☐ Illiterate/Primary ☐ Middle school/High school ☐ University/Postgraduate

Monthly income: ☐ Low income ☐ Moderate income ☐ High income

Smoking status: ☐ Yes ☐ No ☐ Ex-smoker

Medical history: ☐ Hypertension ☐ Diabetes ☐ High cholesterol ☐ Ischemic heart disease ☐ None ☐ Other: ________

Family history of cardiovascular disease: ☐ No ☐ Yes

Knowledge

(Answer: True / False / I don’t know)

Heart and blood vessel disease is the leading cause of death in the world.

Adequate exercise can prevent heart and blood vessel disease.

Eating fruits or vegetables is able to prevent heart and blood vessel disease.

Most heart and blood vessel disease cases are hereditary.

Most heart and blood vessel diseases occur in old people.

Controlling high-fat foods can prevent heart and blood vessel disease.

Stop smoking can prevent heart and blood vessel disease.

Doing housework as an exercise is enough for a day.

Cardiovascular disease can occur in young people.

Obesity can cause heart and blood vessel disease.

Beliefs

(Answer: Agree / Disagree / Uncertain)

Having diabetes increases the risk of heart and blood vessel disease.

Having high blood pressure increases the risk of heart and blood vessel disease.

Having high cholesterol in blood can increase the risk of heart and blood vessel disease.

Taking a daily aspirin when indicated could reduce the chances of heart attack.

High stress in life can cause heart and blood vessel disease.

Use of contraception can increase risk of heart and blood vessel disease.

Attitude

(Answer: Strongly Disagree / Disagree / Uncertain / Agree / Strongly Agree)

I should be doing exercise to maintain a healthy lifestyle.

I should take less saturated oily food and salt for a healthy lifestyle.

I prefer to play with my laptop instead of doing exercise.

I choose to eat or buy fast food when going out with friends.

I am willing to reduce the amount that I eat to improve my health.

I should avoid drinking carbonated drinks.

I believe walking can give benefits to my health.

I should take fruit and vegetables in my diet to maintain my health.

I should control my stress to avoid getting any disease.

Practice

(Answer: Never / Seldom / Frequently / Always)

Does your daily activity involve vigorous activity?

Do you walk for at least 10 minutes to get to and from places?

Do you spend time to exercise at least 20 minutes per day?

How often do you take fruits and vegetables in your diet?

How often do you eat fast food?

Do you take fried food as your main course?

Do you like to eat in between main meals? (snacking)

Do you lead a stressful life?

If you are overweight, have you ever tried to lose weight?

Health Behavior

Do you limit the amount of salt you use in your food, so you don’t develop health problems? ☐ Yes ☐ No

Do you go to the doctor regularly for health checkups? ☐ Yes ☐ No

Where did you get knowledge on CVD? (you can choose more than one)

☐ Physician

☐ Nurse

☐ TV

☐ Internet

☐ Family/Friends

☐ Magazines

Note

This validated survey was taken from a similar study done in the UAE [14].
